# Cannabis and pathologies in dogs and cats: first survey of phytocannabinoid use in veterinary medicine in Argentina

**DOI:** 10.1186/s42238-023-00209-5

**Published:** 2023-11-29

**Authors:** Diana Banach, Paola Ferrero

**Affiliations:** 1 Argentinian Cannabis Veterinarians, Buenos Aires, Argentina; 2 RACME: Committee of Veterinary, Buenos Aires, Argentina; 3 Centro de Investigaciones Cardiovasculares “Dr. Horacio E Cingolani” Facultad de Ciencias Médicas CONICET/UNLP, La Plata, Argentina; 4 Dto de Ciencias Básicas y Experimentales, UNNOBA, Pergamino, Argentina

**Keywords:** Cannabis, Endocannabinoid system, Cats, Dogs

## Abstract

**Background:**

In animals, the endocannabinoid system regulates multiple physiological functions. Like humans, animals respond to preparations containing phytocannabinoids for treating several conditions. In Argentina, laws 27350 and 27669 have expanded the possibility of studying beneficial and adverse effects.

**Materials and methods:**

We conducted a web-based survey of Argentinian Cannabis Veterinarians to make a situational diagnosis on the number of veterinary medicine professionals currently developing treatments with cannabinoids focusing on dogs and cats.

**Results:**

Among the species treated, 77% corresponded to dogs, while 21% were cats. Pain, seizures, and behavior disorders are the most prevalent conditions in dogs. Seven conditions and combinations were treated in cats. Full-spectrum cannabis extract derived from three different chemotypes was administered alone or with standard medication. Response to cannabis treatment was characterized based on improvement categorized according to clinical assessment. Both dogs and cats showed different improvement grades in clinical signs.

**Conclusion:**

This analysis provides promising results regarding the medicinal use of cannabis in dogs and cats. Based on this analysis, we propose to expand the training of professionals, obtain quality preparations, and initiate controlled trials to reinforce knowledge of the use of cannabinoids in veterinary medicine.

**Supplementary Information:**

The online version contains supplementary material available at 10.1186/s42238-023-00209-5.

## Introduction

The endocannabinoid system (ECS), composed of endogen ligands, receptors, and enzymes responsible for their synthesis and degradation, is present in numerous animal species (Silver, 2019; Ritter et al., 2020). The ECS regulates sleep, appetite, behavior, and multiple metabolic functions (Hazzah et al., 2020). Natural molecules and compounds synthesized in the laboratory can bind to ECS receptors, activate signaling pathways, and induce physiological effects mediated by this system in animal species (Schurman et al., 2020).

The ECS plays a role in pathologies that affect other organs and systems, trying to restore homeostasis. Moreover, the ECS can also be dysregulated. Using phytocannabinoids in veterinary medicine may help alleviate symptoms and modify subcellular mechanisms associated with different animal disorders (Hazzah et al., 2020). First, it is essential to understand how it works and in which situations it is deregulated to develop the proper use of phytocannabinoids or cannabimimetics. Furthermore, legal difficulties surrounding the use of cannabinoids in different countries have been a challenge to generating scientific evidence on cannabinoids in veterinary medicine.

In the European Union (EU), the use of cannabis and its derivatives for veterinary purposes is not currently regulated. However, in some EU countries, veterinarians may use cannabis products authorized for human use “off-label” in animals. In the United States of America (USA), medical and recreational cannabis is still illegal under federal law, and veterinarians cannot legally prescribe cannabis for animals. Still, they can guide pet owners who consider using it or potentially could test cannabis products authorized for human use “off-label” in animals, following regulations and guidelines. In Canada, cannabis for medical and recreational use was legalized in 2018. Like in the USA, veterinarians cannot legally prescribe cannabis. The Canadian Veterinary Medical Association (CVMA) has issued guidelines for veterinarians on the use of cannabis in animals. In Japan, cannabis and its derivatives are strictly prohibited, and possession or use can result in severe penalties, including imprisonment. The use of cannabis and its derivatives in veterinary medicine is not allowed (De Briyne et al., 2021).

Despite this issue, their use in dogs, cats, and horses has been reported (Hazzah et al., 2020). Benefits observed of phytocannabinoids and cannabimimetics include reduction in anxiety and pain, improvement in mobility in animals with osteoarthritis, regulation of appetite, control of type 2 diabetes, inflammatory conditions, and epileptic episodes (Hartsel et al., 2019; RC Coelho et al., 2021; Miranda-Cortés et al., 2023). In the example, studies conducted by researchers from the universities of Colorado, Cornell, and North Carolina in the USA have reported data on the pharmacokinetics, safety, and efficacy of CBD in dogs with osteoarthritis as in a group with refractory epilepsy. They explored different routes of administration and parameters associated with safety through analysis of liver enzymes and possible side effects such as diarrhea, eye and nasal secretions, among other evaluations (Hartsel et al., 2019). Other studies have explored, through surveys directed to animal owners, why they bought products with cannabinoids, what types of products they were, and their perception of the animals’ health when they consumed these compounds (Corsato Alvarenga et al., 2023). Most people buy cannabinoid extracts for their dogs to relieve symptoms associated with pathologies such as epilepsy, cancer, anxiety, or arthritis. A small fraction bought cannabinoids for their cats with cancer, anxiety, or arthritis. Benefits are not universal; doses must be carefully adjusted according to each animal’s size, metabolism, and pathology complexity. Differences in the distribution of ECS components will influence treatment outcomes depending on the species and other physical/environmental factors specific to each patient (Hartsel et al., 2019; Pertwee, 2001). Based on these data, controlled studies are necessary to corroborate the effects of cannabinoids on dogs and cats (Kogan et al., 2016).

Due to the modification of laws and regulations in different countries, veterinarians, veterinary medicine researchers, and caregivers agree to approach cannabinoid-based treatments within the framework of generated knowledge that provides conditions of safety and efficacy for patients.

In Argentina, scientific studies on the use of cannabinoids, mainly focused on clinical trials in veterinary medicine, are scarce. We hypothesize that using phytocannabinoids could improve patients’ quality of life with several pathologies, alone or combined with conventional treatments. This survey aims to know the status of the use of cannabinoids in Argentina in veterinary medicine. One objective is to analyze the distribution of the professionals who addressed treatments throughout the country and which species were treated. From this knowledge, we will focus on those treatments performed in dogs and cats. Results are oriented to provide an image of the situation, build scientific evidence, and generate knowledge to provide adequate supervision to caregivers throughout treatment involving cannabinoids for medical use.

## Materials and methods

Between May and November 2021, we conducted the first national survey organized by Argentinian Cannabis Veterinarians. This community groups more than 430 veterinarians nationwide who approach treatments with cannabinoids in multiple pathologies and species. The survey was implemented using a Google Forms hosted in Google Drive and shared between veterinarians following ethical guidelines about the information provided. The questionnaire was structured, self-administered, and categorized as single or multiple choices and with open-field responses. The survey contemplated the following aspects to be evaluated: location/province of the practitioner; species treated; breed; age; weight; pathology; type of feeding; treatments implemented; chemotype of cannabis oil used; the concentration of cannabinoids, route, and dose administered; and the response observed. For analysis, we included dogs and cats with a precise diagnosis who received cannabis oil of known and verified chemotype and presented professional follow-up of the treatment for at least 15 days. The diagnosis of the pathologies was established on the clinical signs identified by the veterinarian at the consultation. In some cases, this diagnosis was confirmed by complementary techniques such as laboratory analysis, x-rays, echocardiography, cytological analysis, and magnetic resonance imaging. The patients were treated with cannabis preparations as adjuvant for the conventional medication or monotherapy. The analysis of the response to treatment was only based on clinical signs observed in consultation and the owners’ report on the animals’ behaviors, which are referred to as symptoms (Rijnberk & Teske, 2009). We do not include information about other species, dogs, and cats treated only once with cannabis, conditions that were not clearly defined, and pregnant females.

Data were collected in a pivot table and plotted in Microsoft Excel v2111. The map showing the density of professionals who responded to the survey was made through the online tool https://paintmaps.com/map-charts/. We made the Sankey plots with SankeyMATIC: https://sankeymatic.com. We used GraphPad v6 as a statistical program. A two-sided Student test was applied to compare doses administered. A comparison of categorical variables was done by *χ*^2^ analysis. In all cases, *p* < 0.05 was considered significant.

## Results

**Fig. 1:**
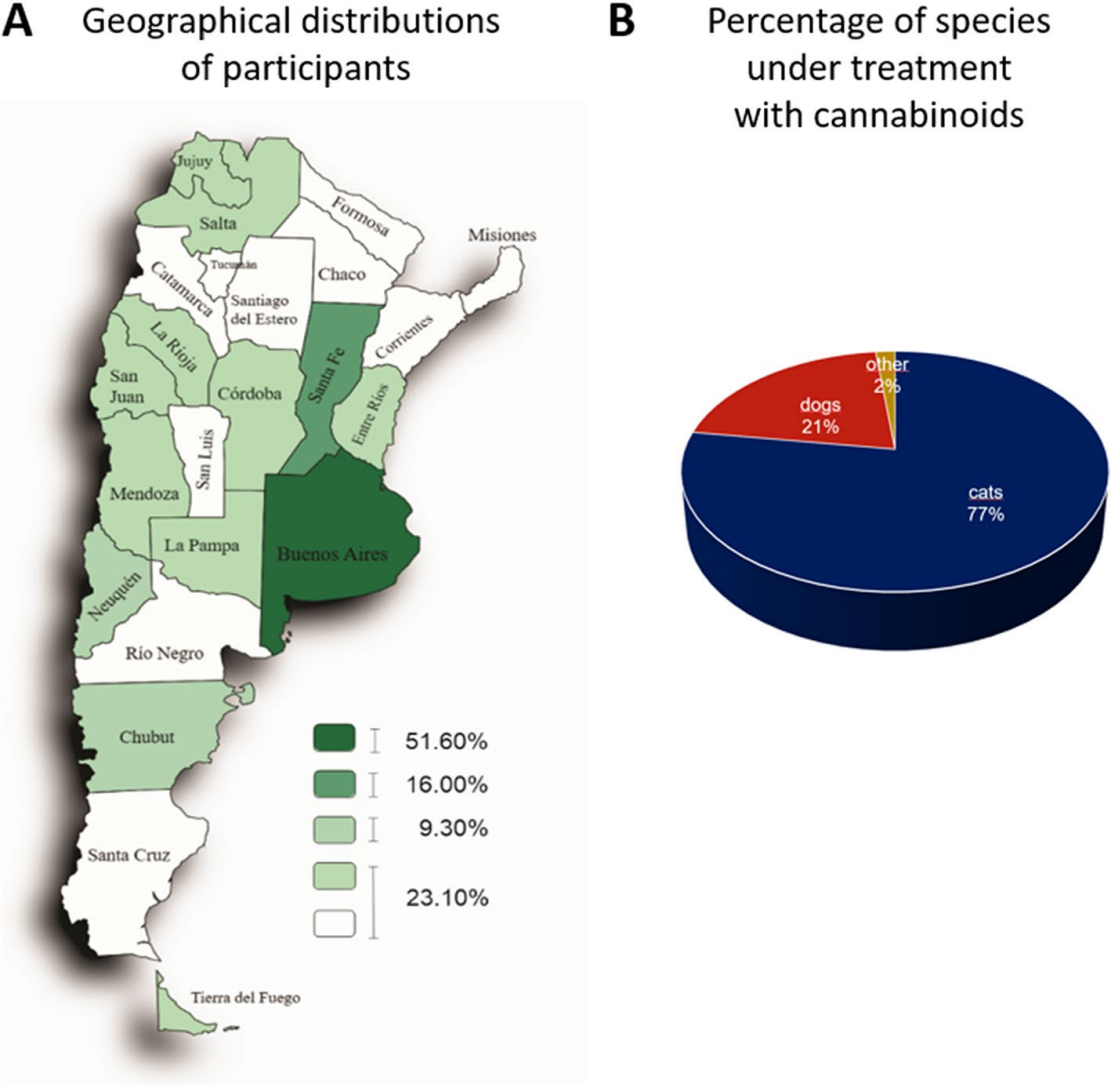
Participants of this survey and treated species. **A** Geographical distribution of veterinarians who participated. The density map shows the geographical distribution of professionals who conduct treatments using cannabinoids. Buenos Aires (51.60%) and Santa Fe (16.00%) concentrate the most significant number of professionals. CABA and Chubut account for 9.30% and the rest of the country 23.10%. Percentage of species under treatment with cannabinoids. **B** The pie chart shows that dogs compound 77% (*n* = 89), cats 21% (*n* = 24), and other species 2% (*n* = 2) of the total of 115 answers

Analysis of the distribution of professionals in medicine veterinary along the country indicated that the highest percentage of veterinarians was concentrated in the provinces of Buenos Aires (51.60%) and Santa Fe (16%), followed by the Autonomous City of Buenos Aires (CABA) (9.30%) and the rest of the provinces (23.10%) (Fig. 1A). Sixty-seven percent of all professionals consulted mentioned that they were small animal generalists, including dogs and cats. Instead, 33% had different specialties such as nutrition, behavioral, ozone therapy, neural therapy, phytotherapy, surgery, nephrology, cat medicine, neurology, physiotherapy, anesthesiology, acupuncture, emergency medicine, and cardiology.

Figure 1B shows that 77% of treated species corresponded to dogs (*n* = 89), 21% to cats (*n* = 24), and 2% to other species (one horse and one turtle). Following, we focused on dogs and cats. In the group of dogs, the most frequent breeds were German Shepherd, Labrador, Dachshund, French Bulldog, Beagle, Cocker, and mixed breeds. In the group of cats, the European Shorthair cat and the Persian and Siamese breeds were the most treated. Both dogs and cats were grouped according to age and the conditions they had. Diseases/conditions were identified through a precise diagnosis based on correct anamnesis and additional tests such as x-rays, laboratory tests, magnetic resonance imaging, and cytology.

**Fig. 2:**
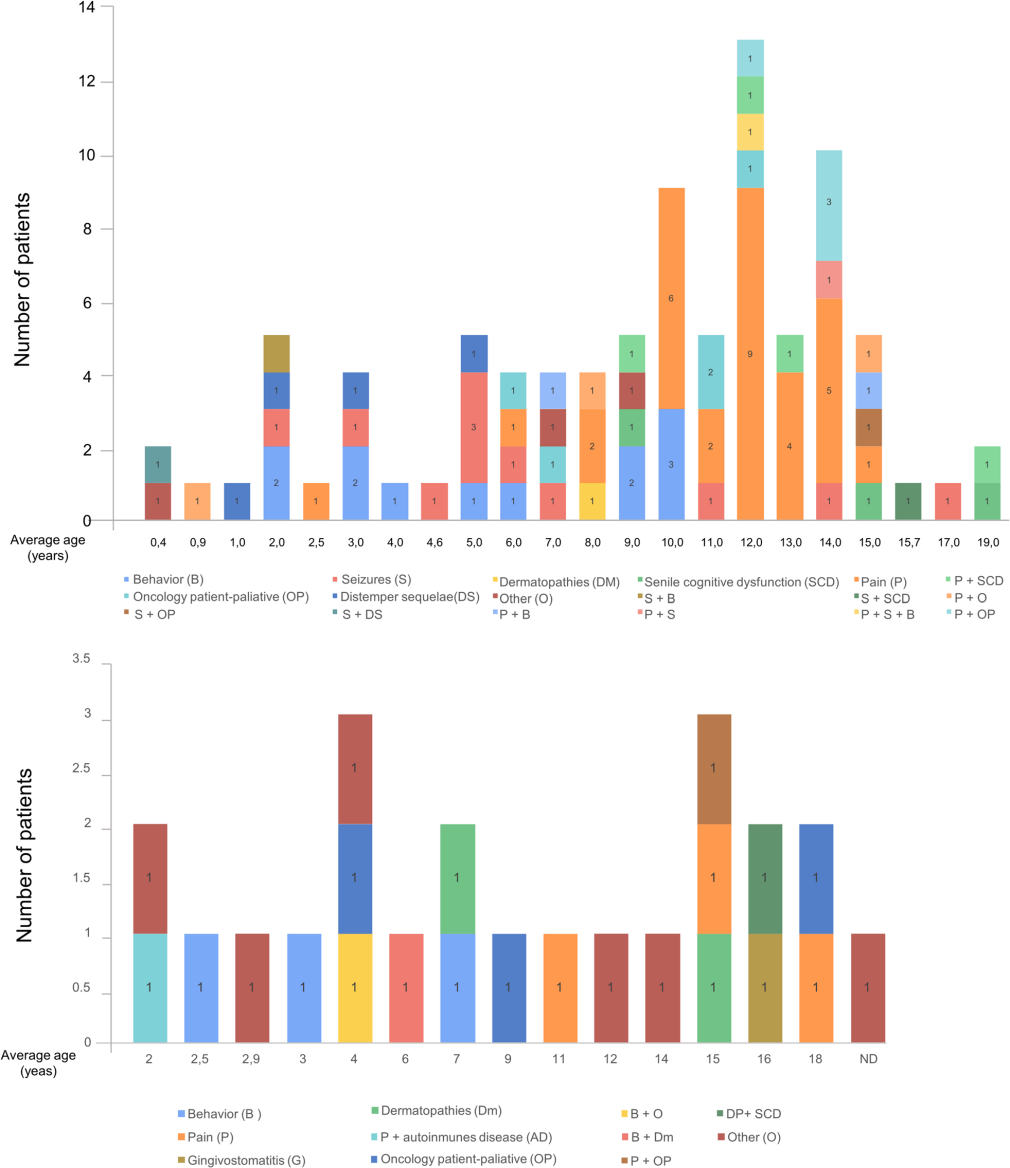
Pathologies identified in dogs and cats. The graphic represents the distribution of the patients according to their age expressed in years. For each age, bars exhibit the number of patients with one or a combination of pathologies/conditions. P, pain; B, behavior; S, seizures; OP, oncology patient; DS, distemper sequelae; DM, dermatopathies; SCD, senile cognitive dysfunction; AD, autoimmune disease; G, gingivostomatitis; ND, non-determined age

The upper graph in Fig. 2 shows that a higher proportion of dogs (*n* = 47) corresponded to animals between 9 and 14 years old. In this group, pain was the most prevalent symptom treated with phytocannabinoids (31 cases). Following that, behavioral disorders (12 cases) and seizures (*n* = 11) were the most frequent conditions. The remaining disorders/diseases included sequelae of distemper, cancer, senile cognitive dysfunction, dermatopathies, and combinations of two or more conditions. The term “other” included not listed pathologies/conditions in the structured question like obesity, arthrosis, tracheal collapse, intrahepatic shunt and metabolic disorder, and nervous and articular signs of leishmania infection.

Cats received in consultation (24 animals) were distributed over a wide age range similar to dogs (2–18 vs. 0.4–19 years, respectively) (Fig. 2, bottom graph). The diseases and conditions observed included behavioral disorders, pain, gingivostomatitis, dermatopathies, autoimmune diseases, and combinations of two or more conditions. Patients with asthma, idiopathic cystitis, and feline immunodeficiency virus were included in the term “other.” Since the number of patients was lower than in the dog group, there were no differences in the number of cases according to the disease/condition treated. However, pain, behavioral disorders, and cancer had three cases each.

**Fig. 3:**
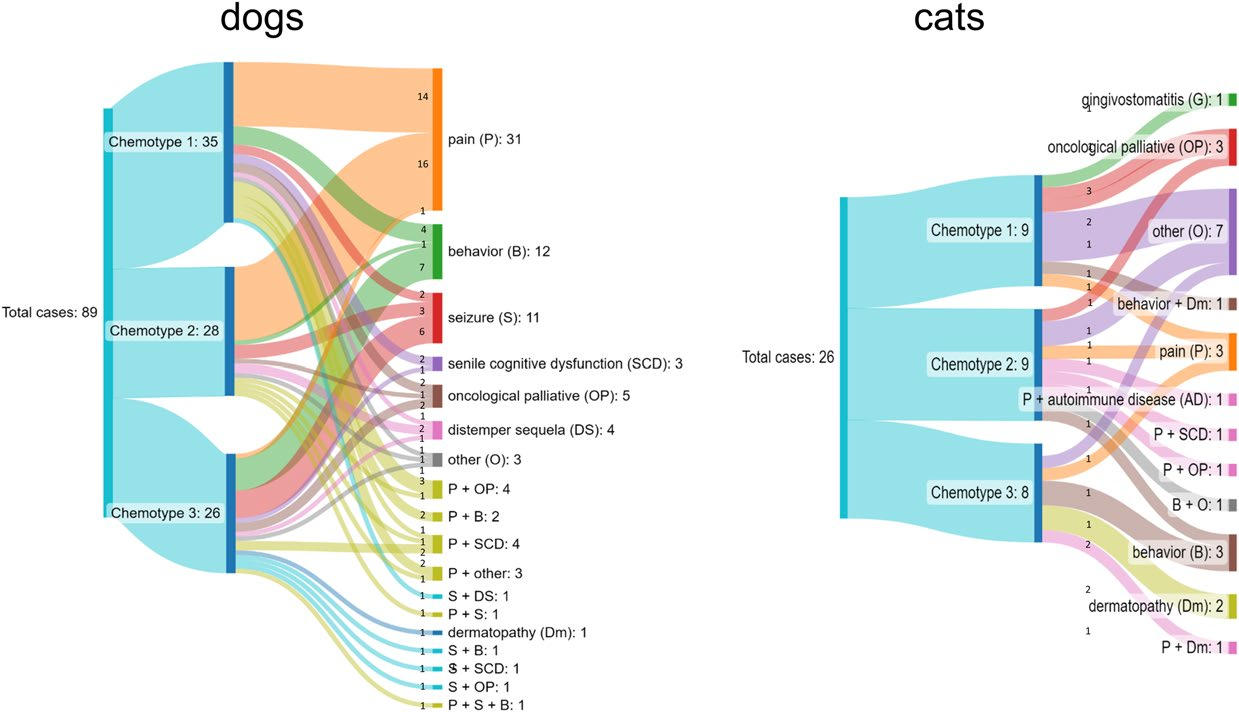
Chemotypes used for each pathology/condition in dogs and cats. Sankey plots show the number of patients treated with chemotypes 1, 2, and 3 and the condition for each chemotype used in both animal groups

Full-spectrum cannabis extract derived from three different chemotypes was utilized. Chemotype 1 contained a higher THC concentration than CBD ([THC] > [CBD]), chemotype 2 was characterized by a similar concentration of both cannabinoids ([THC] = [CBD]), and chemotype 3 presented more CBD than THC ([THC] < [CBD]). Figure 3 shows chemotypes used to treat the different diseases for dogs (left) and cats (right). The route of administration was oral transmucosal.

In dogs, 97% of patients with pain were treated with chemotypes 1 and 2 (45%, *n* = 14 and 52%, *n* = 16, respectively), while chemotype 3 was less used (3% of patients, *n* = 1). Doses were adjusted throughout treatment in order to observe improvement in clinical signs. The dose range was between 1 and 18 drops/day for chemotype 1, with average values between 6 and 7 drops from the beginning of treatment to 60 days. For chemotype 2, the dose range was between 1 and 12 drops/day, with mean values between 3 and 6 drops. On the other hand, 58% of dogs (*n* = 7) with behavioral disorders received chemotype 3. Chemotype 1 was used in 33% (*n* = 4) and chemotype 2 in 8% of patients (*n* = 1) with this condition. Chemotype 3 was also utilized in 54% of patients with seizures (*n* = 6). The dose range of chemotype 3 was between 2 and 10 drops/day with mean values of 4 to 6 drops. In cats, chemotypes 1 and 2 were used for various conditions. Behavioral disorders (*n* = 2) and dermatopathies (*n* = 2) were preferentially treated with chemotype 3. The dose range for all chemotypes was between one and four drops/day with mean values of two drops.

Among the dogs, a subgroup received previously analyzed oil, and the concentrations of the most relevant cannabinoids were determined by chromatography. Table 1 summarizes the initial daily dose of THC and CBD received by patients treated with these oils, adjusted according to the weight of each individual. The conditions with the highest prevalence and the most commonly used chemotype are shown. For example, of the 31 patients with pain, 14 had been treated with chemotype 1, and 10 received one analyzed oil. The mean dose/kg/day for this group of patients was *THC*: 0.083 ± 0.037 and *CBD*: 0.025 ± 0.018. In addition, the table indicates the doses of chemotype 2 used for pain and chemotype 3 for behavioral disorders and seizures. The dosage was adjusted by increasing or decreasing the number of drops/day throughout the treatment.

**Table 1:** Initial doses of THC and CBD administered to dogs and cats

**Dogs**				
	**Chemotype 1**	**Chemotype 2**	**Chemotype 3**	
	**Pain**		**Behavioral disorders**	**Seizures**
**[THC]**^a^ **mg/kg/day**	**0.083 ± 0.0373**	**0.016 ± 0.0064**	**0.007 ± 0.0030**	**0.012 ± 0.0045**
**[CBD]**^**b**^ **mg/kg/day**	**0.025 ± 0.0179**	**0.016 ± 0.0063**	**0.046 ± 0.0110**	**0.048 ± 0.0151**
**n**	**10**	**9**	**7**	**4**
**Cats**				
	**Chemotype 1**	**Chemotype 2**	**Chemotype 3**	
**[THC]**^**a**^ **mg/kg/day**	**0.165 ± 0.0562**	**0.036 ± 0.0134**	**0.061 ± 0.0232**	
**[CBD]**^**b**^ **mg/kg/day**	**0.036 ± 0.0155**	**0.040 ± 0.0163**	**0.383 ± 0.2578**	
**n**	7	**6**	4	

*THC* tetrahydrocannabinol and *CBD* cannabidiol concentrations in chemotypes 1, 2, and 3 preparations. Doses are expressed in mg/kg/day

^a^Concentration of THC

^b^Concentration of CBD

Response to the treatment was only based on the clinical signs observed in consultation by the veterinarian and the information reported by the owner as symptoms. Column 2 of Table 2 shows signs and complementary diagnostic tests utilized for diagnosis, while columns 3 and 4 summarize signs/symptoms evaluated during the treatment.

**Table 2:** Clinical signs and complementary methods used for diagnosis and improvement criteria during cannabinoid treatments

Condition/disease	Additional techniques combined with anamnesis for diagnosis	Signs associated with mild/moderate improvement	Signs associated with significant improvement
Pain (P)	X-ray/echography	Decrease in claudication, recovery of appetite, decrease in pain on palpation	Recovery of functions lost or significantly diminished by the underlying pathology (e.g., playing, barking, running, scratching, grooming), improvement of rest during the night, appetite, etc.
Behavioral disorders (B)	Blood test	Decrease in intensity of symptom/sign manifestations (compulsive behavior, restlessness, anxiety state)	Disappearance of symptoms/signs or patients who can lead an everyday life as any other without behavioral disorders
Seizures (S)	Blood test/magnetic resonance imaging	Decrease seizures’ frequency, duration, or intensity by 10–20%	30% or more decrease in frequency, duration, or intensity of seizures
Oncology patient palliative (OP)	Blood test/echography	Reduction of discomfort but does not restore the quality of life of a non-oncologic patient	Improved appetite, no pain, and mood markedly similar to pre-diagnosis
Distemper sequelae (SM)	Blood test	Decrease in myoclonus/seizures	Disappearance of myoclonus/seizures
Senile cognitive dysfunction (SCD)	Blood test	Decreased vocalization, partial regularization of the sleep-wake cycle, location in space, and interaction with the environment close to normal	Complete recovery of the sleep cycle, the absence of vocalizations, normal appetite, located in space, interacting with the environment
Dermatopathies (DM)	Lab/biopsy	Reduction of skin lesions (erythema, pustules, hyperkeratosis, pruritus)	Almost total or total disappearance of cutaneous sinology
Gingivostomatitis (G)	Blood test/cytology	Decreased salivation, less reddening of the oral mucosa, better chewing of foods	No oral lesions or overt pain, normal chewing, no salivation
P + SCD	Blood tests/x-rays	Improved connection with the environment, normal sleep-wake cycle, even with some alteration in vocalization behaviors or disorientation	Recovery of rest, do not vocalize, patients located in the space where they live, and can interact with their environment similarly to what they did before diagnosis
P + OP	Lab/x-rays/echography	Decreased discomfort and improved rest, partial recovery of appetite	No severe discomfort, no pain or mild pain, good quality of sleep, normal appetite

Shows clinical signs evaluated in consultation and complementary tests for diagnosing pathologies in both species (column 2). Columns 3 and 4 summarize signs/symptoms evaluated for the characterization of improvement along the treatment

We collected information on patients who were in treatment for 15, 30, or 60 days at the time of the survey and classified the perception of the clinical status of patients according to indicators established by the professionals in the consultations. Improvements were classified into the following categories: no change, mild, moderate, and significant.

**Fig. 4:**
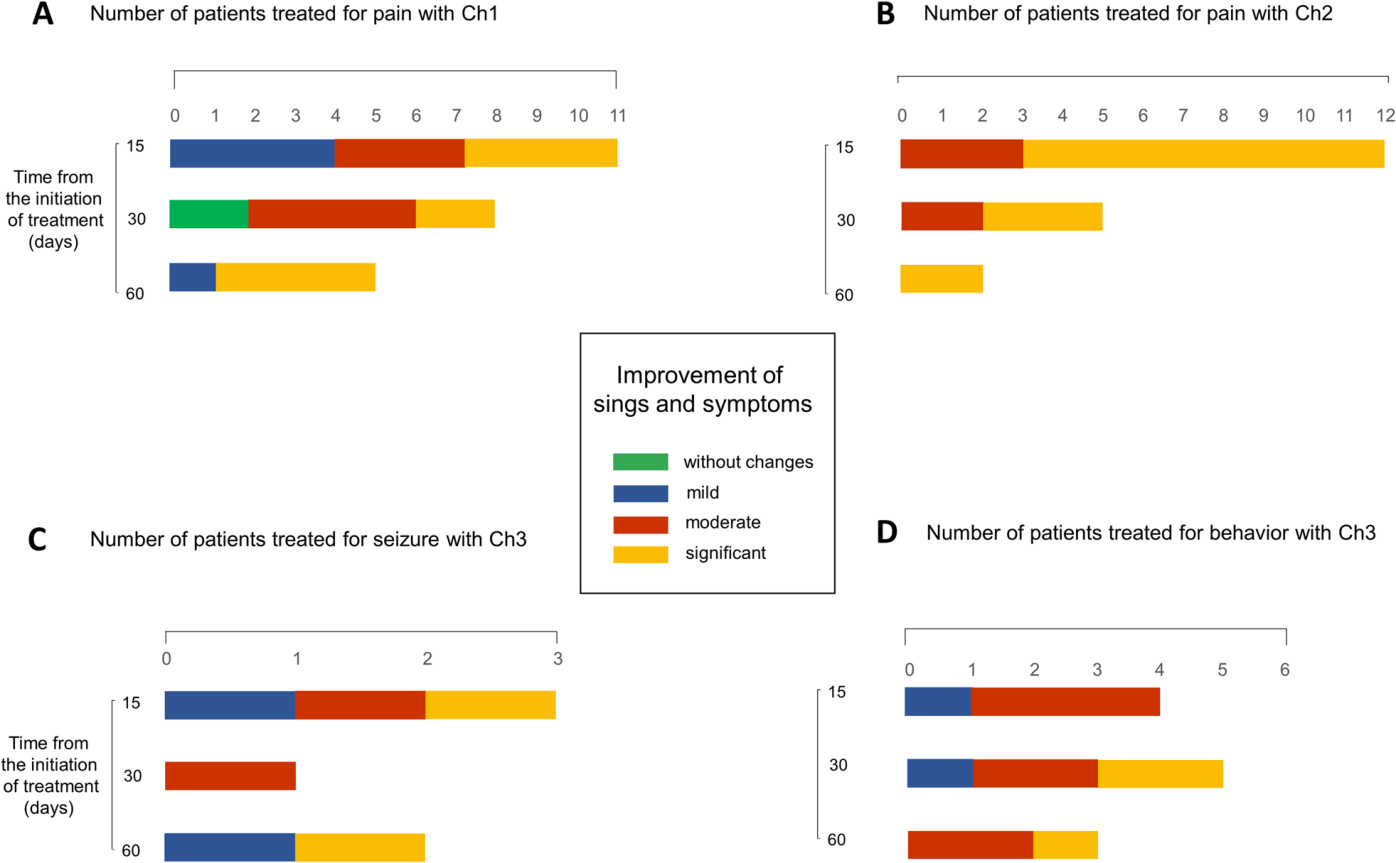
Improvement in dogs along the treatment. The use of cannabinoids alone or combined with other medications improved clinical parameters. A scale was created to classify the improvement in middle, moderate, significant, or without changes. Bars represent the number of patients for each category. More prevalent diseases/conditions and the chemotype (Ch) used are shown. **A** Pain and Ch1. **B** Pain and Ch2. **C** Seizure and CH3. **D** Behavior disorders and Ch3. Patients were evaluated at 15, 30, and 60 days of treatment at the moment of the survey

Among dogs treated for pain with chemotype 1, 36% (*n* = 4) showed mild, 27% (*n* = 3) moderate, and 37% (*n* = 4) significant improvement at 15 days of treatment. At 30 days, 50% (*n* = 4) showed moderate, and at 60 days, 80% (*n* = 4) exhibited significant improvement (Fig. 4A). In the group that received chemotype 2, 75% (*n* = 9) of patients showed significant improvement after 15 days of treatment (Fig. 4B). Those treated for seizures and behavioral disorders with chemotype 3 showed mild, moderate, and significant improvement (Fig. 4C and D). All data correspond to the number of patients evaluated at the moment of the survey. For this reason, the results did not include the complete monitoring of all patients.

For the group of cats, the initial daily doses of THC and CBD for those oils that were analyzed are summarized in Table 1. Chemotypes 1 and 2 were applied for pathologies such as behavioral disorders, pain, and cancer and within the category “other”: gingivostomatitis and asthma. Chemotype 3, in addition to the above, included dermic pathologies.

**Fig. 5:**
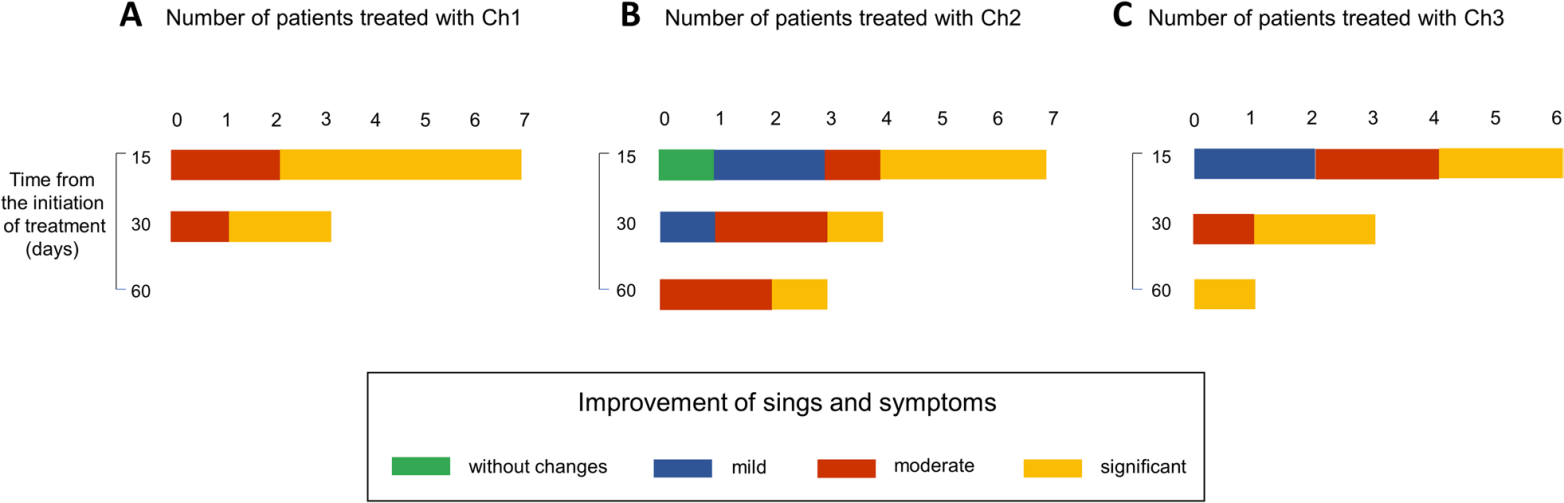
Improvement in cats along the treatment. The use of cannabinoids alone or combined with other medications improved clinical parameters. We classify the improvement in middle, moderate, significant, or without changes. Bars represent the number of patients for each category. Due to that, the number of patients was reduced compared to the dog group, and there is no prevalent pathology. Data were classified according to the chemotype used. **A**, Ch1, **B** Ch2, and **C** CH3. Patients were evaluated at 15, 30, and 60 days of treatment at the moment of the survey

We evaluated the improvement according to the chemotypes used. For chemotype 1, 66% (*n* = 4) showed marked improvement at 15 days (Fig. 5A). For chemotype 2, patients with mild, moderate, and marked improvement were observed, as well as one case with no change (Fig. 5B). For chemotype 3, the percentage of patients with significant improvement was 43% (*n* = 3) at 15 days and 75% (*n* = 3) at 30 days (Fig. 5C). Like in dogs, all data about the cat group correspond to the number of patients evaluated at the moment of the survey.

**Fig. 6:**
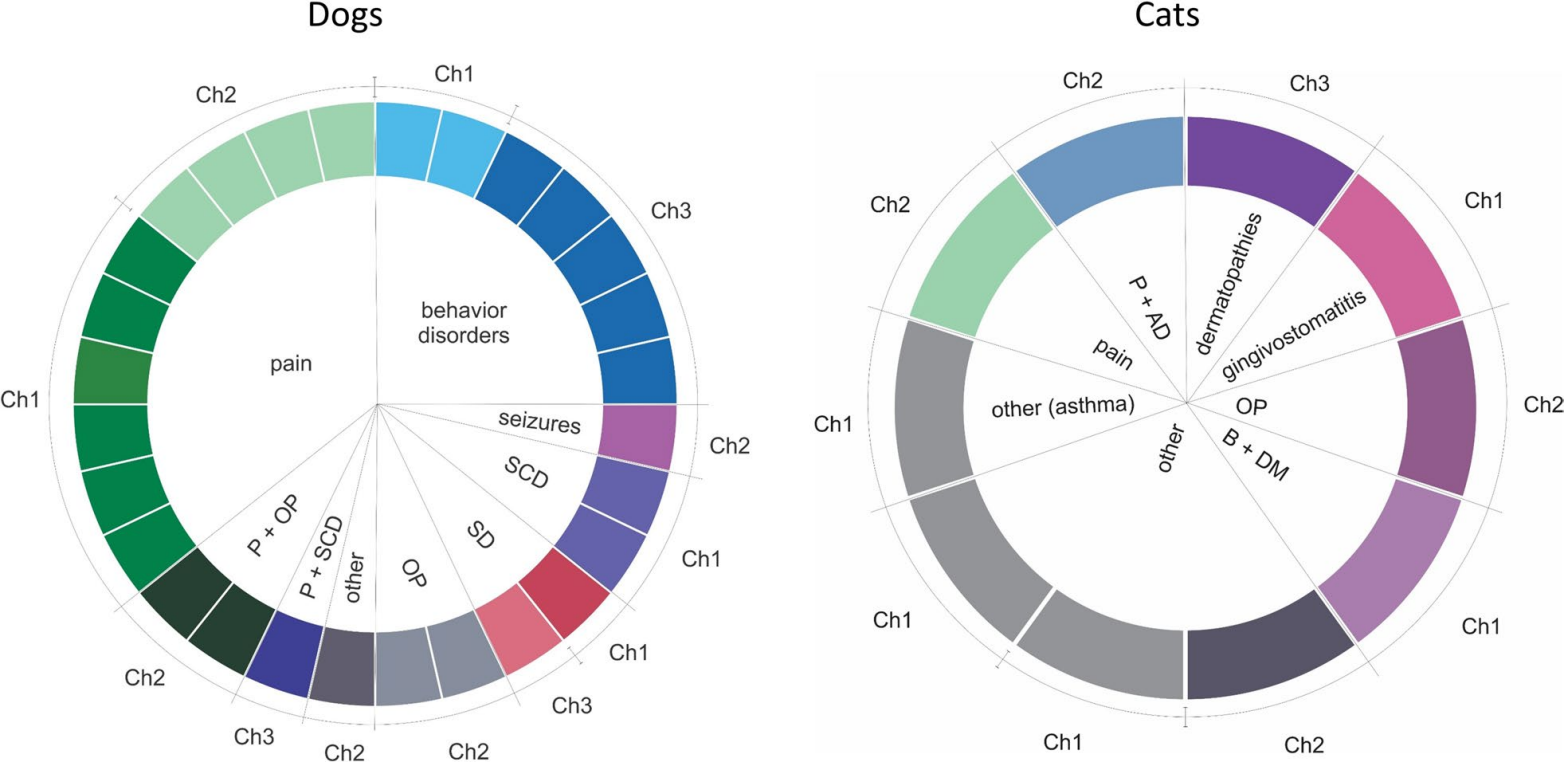
Dogs and cats treated only with cannabinoids. Pie graphs show the number of patients with one or a combination of pathologies and the chemotype used. P, pain; OP, oncologic patient palliative; DS, distemper sequela; SCD, senile cognitive dysfunction; B, behavior disorders; DM, dermatopathy; and AD, autoimmune disease

**Fig. 7:**
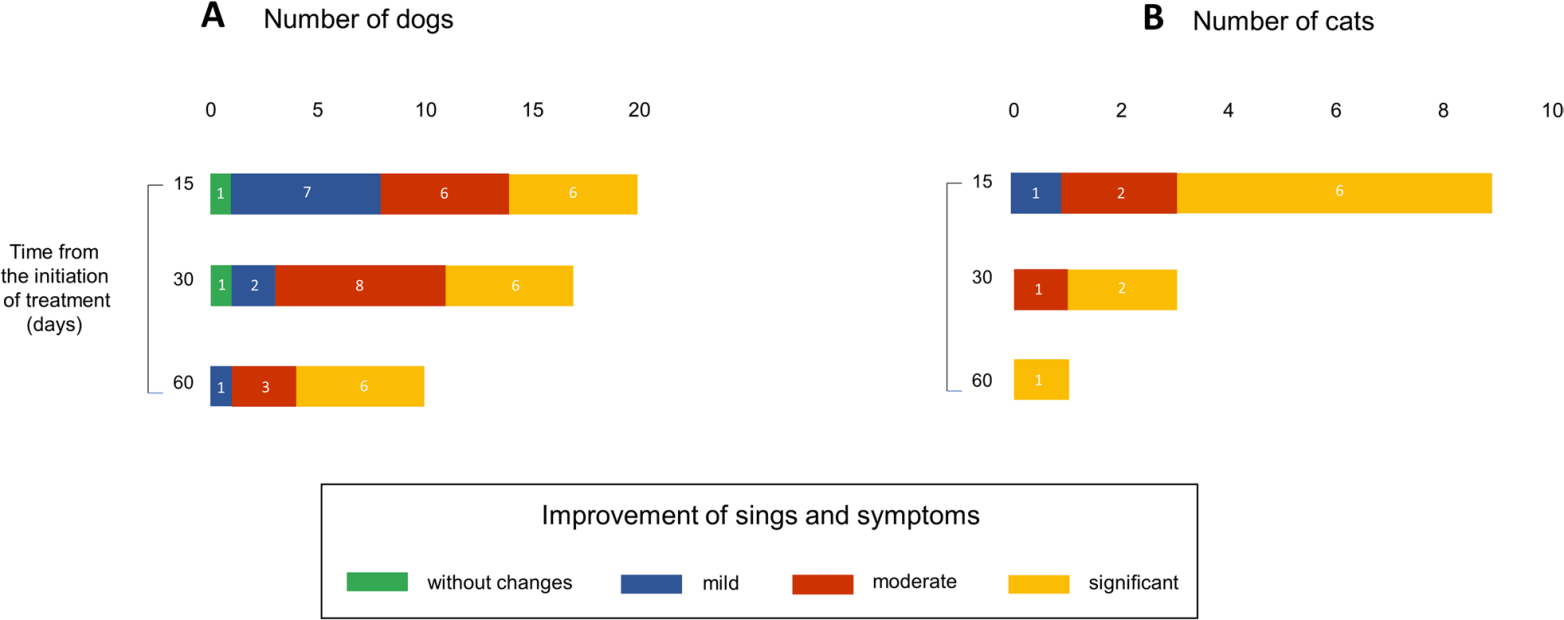
Response of animals treated only with cannabinoids. Improvement is recognized at different times of treatment in patients evaluated. We classify the improvement in middle, moderate, significant, or without changes. Bars represent the number of patients for each category

In the dog group, 28 out of 89 animals received cannabis oil extract as monotherapy. For pain treatment, cannabis as monotherapy was delivered to 10 of the 31 patients with this condition. Eight out of 11 animals received the extract for behavior disorders, and other individual cases were treated only with cannabis oil. For behavior disorders, six of seven animals were given cannabinoids as a single therapy. Figure 6A summarizes data showing which chemotype was used in each case. We compared doses of cannabinoids in polymedicated patients with doses applied to the other group that received it as single therapy. Supplemental Tables 1 and 2 show values for all chemotypes utilized for the most relevant conditions. Doses of cannabinoids were received for polymedicated patients, and the group that received only cannabinoid preparations was not significantly different. In all cases, veterinarians and caregivers reported a positive perception of treatments indicating moderate and significant improvement in the quality of life at 15, 30, and 60 days (Fig. 7A).

In order to evaluate whether chemotype 1 or chemotype 2 was the most appropriate for the treatment of pain, we compared the number of animals that showed significant improvement to those that showed mild/moderate improvement after 15 days treated with chemotypes 1 or 2. The relationship between these variables was not significant, *Χ*^2^ (1, *N* = 23) = 3.4862, *p* = 0.061. Therefore, both chemotypes relieved the symptoms.

The adverse effects reported in dogs were sedation and a case of a paranoid state in a poly-medicated patient with a behavioral disorder. The veterinarian replaced the oil with another one of the same chemotype, and the adverse effect was reversed.

Ten cats received cannabis as a single therapy. Diseases included gingivostomatitis, dermatopathies, oncologic disease, pain, asthma, and signs and symptoms produced by two pathologies. Data are shown in Fig. 6B. Doses of cannabinoids were received for polymedicated patients, and those that received only oil were not significantly different (Supplemental Table 3). Like in the dog’s group, veterinarians and caregivers perceived a reduction of clinical signs and symptoms at 15, 30, and 60 days of treatment (Fig. 7B).

Then, we analyzed whether the type of food influenced the improvement in clinical conditions in both groups. Supplemental Table 4 shows, for dogs and cats, the proportion of animals that presented different grades of improvement related to the total of animals fed using raw food, cooked food, ultra-processed, and mixed diets. In dogs and cats (Table S4), more than 50% of animals showed significant improvement independently of the type of diet. To evaluate whether the type of food was related to the improvement during the treatment, we compared the number of animals that showed mild/moderate or significant improvement according to the food consumed, looking for possible drug-food interaction. The relationship between these variables was not significant, *Χ*^2^ (3, *N* = 89) = 3.2527, *p* = 0.3542 for dogs, and *Χ*^2^ (3, *N* = 24) = 2.7563, *p* = 0.4307 for cats. According to these data, feeding is not associated with the response during the treatment.

## Discussion

In Argentina, recent regulation of Law 27669 allows the production of medical cannabis and hemp (Banach & Ferrero, 2023). This modification of the legal frame permitted veterinarians and owners to look at the potential of cannabis for animal treatments. This survey provides information on the current status of cannabis use in veterinary medicine.

According to previous data from our research group (Banach & Ferrero, 2023), in 16 of the 23 provinces of the country, there were professionals interested in the use of cannabinoids. This interest was more clearly visible in newly licensed veterinarians, as we demonstrated in a previous report (Banach & Ferrero, 2023). In contrast, for example, a survey conducted in the USA showed that young veterinarians were less likely to recommend or prescribe CBD, which was related to the legal status of cannabis use in each state (Kogan et al., 2019).

Here, we evaluated treatment outcomes in dog and cat populations, constituting the most significant percentage of treated species. We found that, in dogs, the predominant condition treated with phytocannabinoids was chronic osteomyoarticular pain, mainly associated with aging. Studies and case reports have shown that pain management is one of the main reasons for incorporating cannabinoids in treatments (Hazzah et al., 2020; Landa et al., 2022). In this survey, the preparations used by practitioners for pain treatment came from chemotypes 1 and 2, with a predominant or balanced concentration of THC over CBD respectively. In other reports, instead, CBD, as a single component or in higher concentration, has been observed to reduce pain and increase activity in dogs in a range of 2 to 50 mg/kg/day during 2 to 12 weeks depending on the study (Di Salvo et al., 2023). Another difference between these reports and our findings lies in the doses reported here in the order of micrograms compared to those used in other studies. However, the analysis of clinical signs allowed reporting a mild/moderate to significant improvement, depending on the case, which was not associated with the chemotype used.

Seizures and behavioral disorders were the other most prevalent conditions in dogs. Using preparations with higher CBD concentration (chemotype 3) reduced seizures’ frequency, duration, and intensity in epileptic conditions and minimized behavioral disorders (anxiety, restlessness, compulsive behaviors), respectively. Other studies have evaluated the clinical efficacy of CBD-based preparations at doses from 1 to 10 mg/kg/day administered orally. In this survey, doses in the order of micrograms result in an improvement in clinical signs. The positive effect of microdoses has not been reported in veterinary medicine. However, its effectiveness has been observed in humans (Ruver-Martins et al., 2022). A possible reason to explain these differences could lie in the concentration and variety of terpenes and other components that could influence the action of cannabinoids through synergistic effect (Sommano et al., 2020), considering that the preparations are full spectrum. Full-spectrum preparations, obtained from the plant through minimal processing, are rich in cannabinoids, terpenes, flavonoids, fatty acids, and other components (Corsato Alvarenga et al., 2023). Another possible cause could be the route of administration which, in the case of oral transmucosal, would partially avoid first-pass metabolism allowing the cannabinoids to reach the systemic circulation. However, a report comparing oral and oral-transmucosal routes of administration showed no differences in CBD bioavailability in dogs (della Rocca et al., 2023).

Among the adverse effects, in this survey, we found one case of excess sedation with chemotype 3 (CBD-predominant oil) and one case of a paranoid state with chemotype 1 (THC-predominant oil); both cases were reported in dogs. Both were reversed by dose adjustment and provision of new preparation. Studies analyzing different doses and preparations with different THC:CBD ratios in dogs indicated that CBD-predominant oils are better tolerated than formulations with higher THC concentrations (Vaughn et al., 2020). In addition, it is relevant to evaluate the possible interactions of cannabinoids with other medications, especially because most patients are polymedicated (Antoniou et al., 2020). Regarding cats, less is known about the effects of cannabinoids. Studies about the pharmacokinetics in healthy cats reported that doses between 2.8 and 30.5 mg/kg (Yu & Rupasinghe, 2021) have been tolerated. However, their beneficial effects on cats’ pathophysiology remain unclear. Although some conclusions obtained in dogs might be extrapolated, it is necessary to consider interspecific differences.

Here, we present an exploratory study to determine the status of cannabinoid use in veterinary medicine. According to these promising results on the use of cannabinoids, it is mandatory to develop a plan to build solid scientific evidence. The following aspects should be included: (1) education of veterinarians about the function and dysregulation of ECS as medicinal properties of cannabinoids; (2) knowing the origin and composition of the preparations, as well as the relative content of the main cannabinoids and other compounds such as terpenes, flavonoids, and heavy metals, before being applied on patients; (3) initiate treatments with the lowest possible doses, according to scientifically based studies, mainly because the distribution and density of cannabinoid receptors are different in dogs and cats compared to humans; and (4) document beneficial and adverse effects, through rigorous clinical trials testing cannabinoids and placebo, as well as individual case reports. Part of these actions are underway, allowing cannabinoid treatments to be safely applied on a case-by-case basis to improve patients’ quality of life.

### Limitations and bias of the study

This survey is the first one conducted in Argentina, and it is exploratory. Possibly, a higher proportion of veterinarians who treat dogs and cats with cannabinoids did not access the online survey. Such restriction might underestimate the number of cases documented. Analysis of cats requires an incremented number of cases to elaborate a robust analysis of the effectiveness of cannabinoids in treatments of the mentioned conditions. This survey presents a situational diagnosis and is not a placebo-controlled study. Analysis of clinical signs not always was standardized for all practitioners. Future studies are needed to analyze specific races and conditions.

### Supplementary Information

**Additional file 1 : Supplemental Table 1.** Doses of main cannabinoids of chemotypes 1 and 2 administered to dogs with pain. **Supplemental Table 2.** Doses of main cannabinoids of chemotype 3 administered to dogs with behavioral disorders. **Supplemental Table 3.** Doses of main cannabinoids of chemotypes 1, 2, and 3 administered to cats. **Supplemental Table 4.** Incidence of food on the response to cannabinoids treatment.

## Data Availability

Supporting data and the questionnaire are available in supplemental data.
