# Supplementary material for: Cannabis and pathologies in dogs and cats: first survey of phytocannabinoid use in veterinary medicine in Argentina
Source: J Cannabis Res. 2023 Nov 29;5:39. doi: 10.1186/s42238-023-00209-5 (PMC10685507; doi:10.1186/s42238-023-00209-5)
Supplement: Supplementary file 1 — Additional file 1 : Supplemental Table 1. Doses of main cannabinoids of chemotypes 1 and 2 administered to dogs with pain. Supplemental Table 2. Doses of main cannabinoids of chemotype 3 administered to dogs with behavioral disorders. Supplemental Table 3. Doses of main cannabinoids of chemotypes 1, 2, and 3 administered to cats. Supplemental Table 4. Incidence of food on the response to cannabinoids treatment. [file 42238_2023_209_MOESM1_ESM.docx]

**Supplemental Table 1: Doses of main cannabinoids of chemotypes 1 and 2 administered to dogs with pain.**

| Dogs - Pain | | | |
| --- | --- | --- | --- |
| Chemotype 1 | | | |
|  | Polimedicated | Only cannabinoids | significance |
| [THC] mg/kg/day | 0.096 ± 0.054 | 0.055 ± 0.011 | NS |
| [CBD] mg/kg/day | 0.035 ± 0.025 | 0.004 ± 0.003 | NS |
| n | 7 | 3 |  |
|  | | | |
| Chemotype 2 | | | |
|  | Polimedicated | Only cannabinoids |  |
| [THC] mg/kg/day | 0.008 ± 0.003 | 0.027 ± 0.013 | NS |
| [CBD] mg/kg/day | 0.007 ± 0.003 | 0.028 ± 0.012 | NS |
| n | 5 | 4 |  |

Extracts from chemotypes 1 and 2 were provided to different groups of dogs as monotherapy or in conjunction with other medication for managing pain. [THC] tetrahydrocannabinol and [CBD] cannabidiol concentrations. The amount is expressed in mg/kg/day. The comparison between poly-medicated animals or those that received only the extract did not provide significant results. Student test p <0.05. NS: non-significant.

**Supplemental Table 2: Doses of main cannabinoids of chemotype 3 administered to dogs with behavioral disorders.**

| Dogs – Behavior disorders - seizures | | | |
| --- | --- | --- | --- |
| Chemotype 3 | | | |
|  | Polimedicated | Only cannabinoids | significance |
| [THC] mg/kg/day | 0.096 ± 0.058 | 0.055 ± 0.011 | NS |
| [CBD] mg/kg/day | 0.035 ± 0.025 | 0.004 ± 0.003 | NS |
| n | 7 | 3 |  |

Extracts from chemotype 3 were provided to different groups of dogs as monotherapy or in conjunction with other medication for managing behavior disorders. [THC] tetrahydrocannabinol and [CBD] cannabidiol concentrations. The amount is expressed in mg/kg/day. The comparison between poly-medicated animals or those that received only the extract did not provide significant results. Student test p <0.05. NS: non-significant.

**Supplemental Table 3: Doses of main cannabinoids of chemotypes 1, 2, and 3 administered to cats.**

| Cats | | | |
| --- | --- | --- | --- |
| Chemotype 1 | | | |
|  | Polimedicated | Only cannabinoids | significance |
| [THC] mg/kg/day | 0.241 ± 0.076 | 0.052 ± 0.023 | NS |
| [CBD] mg/kg/day | 0.041 ± 0.024 | 0.030 ± 0.023 | NS |
| n | 4 | 3 |  |
|  |  |  |  |
| Chemotype 2 | | | |
| [THC] mg/kg/day | 0.052 ± 0.025 | 0.021 ± 0.004 | NS |
| [CBD] mg/kg/day | 0.061 ± 0.029 | 0.019 ± 0.003 | NS |
| n | 3 | 3 |  |
|  |  |  |  |
| Chemotype 3 | | | |
| [THC] mg/kg/day | 0.052 ± 0.025 | 0.025 ± 0.000 | - (n too small) |
| [CBD] mg/kg/day | 0.061 ± 0.029 | 1.150 ± 0.000 | - (n too small) |
| n | 3 | 1 |  |

Extracts from chemotypes 1, 2, and 3 were provided to different groups of cats as monotherapy or in conjunction with other medication for managing multiple pathologies, as indicated in Figure 2. [THC] tetrahydrocannabinol and [CBD] cannabidiol concentrations. The amount is expressed in mg/kg/day. The comparison between poly-medicated animals or those that received only the extract did not provide significant results. Student test p <0.05. NS: non-significant.

**Supplemental Table 4: Incidence of food on the response to cannabinoids treatment.**

| Feeding | Dogs | | Cats | |
| --- | --- | --- | --- | --- |
|  | Mild/moderated | Significant | Mild/moderated | Significant |
| Raw food | 1 | 6 | 1 | 1 |
| Cooked food | 1 | 4 | 2 | 2 |
| Ultra-processed | 14 | 26 | 1 | 8 |
| Mixed | 7 | 30 | 3 | 6 |

The table compares the number of dogs and cats with presented mild/moderated versus significant improvement of clinical signs and cannabis treatment grouped according to their feeding. The relationship between these variables was not significant, X2 (3, N = 89) = 3.2527, p = 0.3542 for canines and X2 (3, N = 24) = 2.7563, p = 0.4307 for felines.

**Questionnaire**

Each form corresponds to one patient

1 Email

2 Name of patient

3 Province

4 Specie: canine/feline/other

5 Age

6 Weight

7 Food: raw/cooked/ultra-processed/mixed

8 Pathology: pain/seizures/distemper sequelae/behavioral disorder/autoimmune diseases/gingivostomatitis/dermatopathies/senile cognitive dysfunction/oncologic patient/other

9 Possess definitive diagnosis: yes/no

10 Treatment: polimedicated/only cannabis/complementary therapies

11 Which chemotype of cannabis use: ch1/ch2/ch3

12 Do you use analyzed oil: yes/no

13 If you use analyzed oil, write mg/ml of THC:CBD

14 Indicate dose: drops/day

| Initial | 15 days | 30 days | 60 days |
| --- | --- | --- | --- |

15 Evaluate the improvement of signs according to the following categorization:

| Improvement | 15 days | 30 days | 60 days |
| --- | --- | --- | --- |
| None |  |  |  |
| Mild |  |  |  |
| Moderate |  |  |  |
| Significant |  |  |  |
| Deteriorated |  |  |  |
| Treatment interruption |  |  |  |

16 Adverse effects: yes/no

17 If yes: can you reverse? Yes/no

18 Comments
